# Switch mode capacitive pressure sensors

**DOI:** 10.1038/s41378-022-00469-w

**Published:** 2022-12-23

**Authors:** Nabil Shalabi, Kyle Searles, Kenichi Takahata

**Affiliations:** 1 Department of Electrical and Computer Engineering, University of British Columbia, Vancouver, BC V6T 1Z4 Canada; 2 School of Biomedical Engineering, University of British Columbia, Vancouver, BC V6T 1Z3 Canada

**Keywords:** Electrical and electronic engineering, Sensors

## Abstract

Switch mode capacitive pressure sensors are proposed as a new class of microfabricated devices that transform pressure into a mechanically switching capacitance to form an analog-to-digital signal with zero power, high sensitivity, and a high signal-to-noise ratio. A pressure-sensitive gold membrane suspended over a capacitive cavity makes ohmic contact with patterned gold leads on the substrate, closing circuits to fixed on-chip capacitors outside the cavity and leading to significant step responses. This function is achieved by allocating the switch leads on the part of the counter electrode area, while the remaining area is used for touch mode analog capacitive sensing. The sensor microchip is prototyped through a novel design approach to surface micromachining that integrates micro-Tesla valves for vacuum sealing the sensor cavity, showing an unprecedented response to applied pressure. For a gauge pressure range of 0–120 mmHg, the sensor exhibits an increase of 13.21 pF with resultant switch events, each of which ranges from 2.53–3.96 pF every 12–38 mmHg, in addition to the touch mode linear capacitive increase between switches. The equivalent sensitivity is 80–240 fF/mmHg, which is 11–600× more than commercial and reported touch mode sensors operating in similar pressure ranges. The sensor is further demonstrated for wireless pressure tracking by creating a resonant tank with the sensor, showing a 32.5–101.6 kHz/mmHg sensitivity with frequency jumps led by the switch events. The developed sensor, with its promising performance, offers new application opportunities in a variety of device areas, including health care, robotics, industrial control, and environmental monitoring.

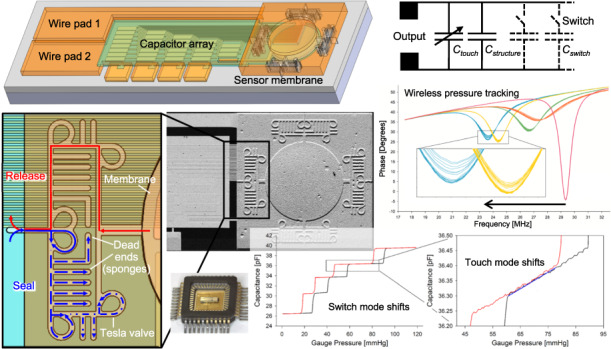

## Introduction

Pressure sensing technology is poised for new innovations in fundamental pressure sensing modalities. Capacitive pressure sensors are now more commercially available owing to their higher sensitivity, low turn-on temperature drift, and robust performance that is less sensitive to environmental conditions compared to their piezoresistive counterpart^1–6^. These advantages motivated a large research effort toward touch mode capacitive pressure sensors that leverage microelectromechanical system (MEMS) technology as a way to further improve sensor sensitivity, linearity, full-scale range, and durability^1–26^. However, we have yet to see MEMS sensing hardware that uses a single device in a pressure sensing mode that transforms continuous analog pressure inputs into mechanically switched digital capacitive outputs throughout the full sensing range with zero power consumption. We refer to this class of sensors as switch mode capacitive pressure sensors, which operate by activating a capacitive switch array using a pressure-sensitive membrane as a contact element. These sensors can solve problems in transducing low-pressure signals or small changes in pressure by producing orders-of-magnitude larger capacitive signals for applications that require high sensitivity or signal-to-noise ratios.

Analog-to-digital (A/D) converters are the cornerstone of much of the technology we use today as they make for signals that are easier to process and more robust to noise^27^. The proposed switch mode pressure sensor is capable of directly converting an analog pressure to a digital capacitance with no additional power or separate A/D chips. These capabilities could uniquely advance a broad range of application areas that need pressure tracking and management, including medical devices, robotics, human interface devices, environmental and industrial pressure alarms, as well as applications that go beyond analog sensing with pressure switching into other circuit elements such as a wake-up circuit in zero power electronics and the Internet of Things. The capacitive principle of the device allows for the implementation of resonant wireless sensing through simple passive circuitry without needing a dedicated power source, which greatly aids the miniaturization of the wireless sensors^28–33^.

The switch mode capacitive pressure sensor is fundamentally driven by advancing the concept of touch mode sensors^1–4^. In touch mode, a pressure-sensitive membrane is used as a deflectable capacitive electrode, where at the operating pressure range, the membrane makes contact with a thin dielectric covering the fixed counter electrode on the sensor’s substrate. As pressure increases, the membrane contact area increases and results in an increase in capacitance. Accordingly, the touch mode sensor is a pressure-dependent capacitor with a variable area, unlike the original capacitive pressure sensor, which depends on small changes in the gap separating the capacitive electrodes^1–4^. Although touch mode sensors offer various improvements, as noted earlier^1–4^, their core performance, including the sensitivity and full-scale range, is still inherently limited by the area and mechanics of the capacitive membrane, which also poses challenges in miniaturization for applications that require high capacitance. The proposed switch mode sensor overcomes the touch mode’s fundamental drawbacks by placing conductive leads in the path of the membrane’s expected contact area on the substrate, where ohmic contact with every lead closes a circuit to an on-chip fixed capacitor or other circuitry connected outside the membrane area.

Medical devices are one of the application areas that significantly benefit from this type of pressure sensor. Many electronic “smart” implants aim to track local pressure in vivo and transmit the data, typically through radiofrequency (RF) waves, to the outside of the body for diagnostic and therapeutic purposes^34^. Stents, or tubular scaffolds implanted to open diseased or narrowed ducts inside the body, are an exemplary device area for this technology. Smart vascular and ureteral stents have been reported for RF sensing of blood and kidney pressures using integrated MEMS capacitive sensors for wireless diagnosis of their target diseases (known as restenosis and hydronephrosis, respectively)^28,31–33^. The proposed switch mode sensor allows smart implants to automatically issue an alert when the local pressure reaches a dangerous threshold level, thereby enabling immediate necessary treatments. Signal damping and noise in RF communication with a device inside the body remains a challenge, particularly in resolving small pressure/capacitance changes when using conventional analog sensors^28,31–33^. This can be effectively addressed by a switch mode sensor that provides distinct step increases of capacitance at designed pressure levels, which, in turn, vastly raises the sensitivity and reliability of sensing through the implant. The proposed sensor is expected to bring similar benefits to a variety of application fields far beyond smart implants and medical devices.

In this paper, we elaborate on the concept behind the switch mode sensor as well as the integration of this concept into a hybrid device that combines the switch mode sensor with a touch mode sensor. Here, we present the design of a proof-of-concept prototype and further show a novel process flow to surface micromachine the sensors with vacuum packaging by vapor-phase polymer deposition using integrated one-way microscale valves based on the concept of a Tesla valve^35–38^, which uniquely enables ohmic contact switching with the deflected membrane inside the vacuum-sealed cavity. We then show the experimental results and detailed electromechanical analysis of the sensor prototype. Finally, the switch mode sensor is coupled with an inductive coil antenna for an experimental demonstration of resonance-based wireless pressure tracking.

## Working principle and design

**Fig. 1:**
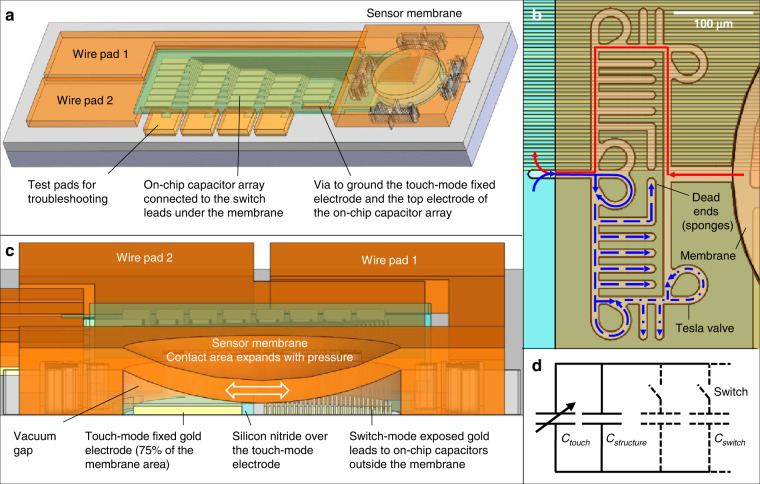
Design and architecture of the switch mode capacitive pressure sensor. **a** Three-dimensional (3D) view of the device highlighting the sensor membrane, on-chip switch capacitor array, and wiring/test pads. **b** Top view of the release and sealing channel with integrated micro-Tesla valves. The red line indicates the path used to release the sacrificial layer under the membrane, and the blue lines indicate the path to the cavity taken by Parylene molecules where their forward flow is reduced after passing each Tesla valve (dashed blue lines) and finally blocked (the red and blue fluxes are illustrated only on either side of the symmetric channel pattern, but each occurs on both sides). **c** Cross-sectional side-view of the sensor showing the vacuum cavity with the membrane deflected under atmospheric pressure (out-of-plane dimensions are not drawn to scale). The illustration highlights the internal sensor layers as well as the expected movement and increase in the contact area as the applied pressure increases. **d** An equivalent circuit of the sensor showing the touch mode variable capacitance (*C*_touch_), parasitic capacitance (*C*_structu*re*_) from the frame structural parts of the sensor, and an array of the switch capacitances (*C*_switch_, shown with dashed lines) that are consecutively switched upon pressure increase to drastically raise the sensor’s total capacitance stepwise.

The targeted capacitive pressure sensor combines analog signals from a *touch mode* variable capacitor with digital signals from *switch mode* discrete capacitors that are mechanically activated as a pressure-deflected membrane makes ohmic contact with an array of relays connected to on-chip fixed capacitors (Fig. 1a). This absolute pressure sensor operates with the mechanics of a movable gold membrane that plays a dual role as the variable capacitive electrode and the contact relay electrode, both driven by ambient pressure. The membrane is suspended over a capacitive cavity that is vacuum sealed through passive check valves known as Tesla valves^35–38^ integrated into the cavity’s release/seal channels (Fig. 1b). At atmospheric pressure, the membrane deflects and makes contact with an insulated counter electrode (silicon nitride-coated gold) fixed on the substrate. As ambient pressure changes, the contact area varies accordingly (Fig. 1c), resulting in corresponding changes in the touch mode capacitance^1–4^. This fixed electrode is designed to occupy ~3/4 of the membrane area. In the remaining 1/4 of the area, an array of exposed gold switch leads are uniformly patterned and connected to fixed capacitors (silicon nitride sandwiched between gold thin films) integrated outside the cavity. As the membrane contact area expands with pressure, this results in consecutive contact with the patterned leads, causing switch events that add the on-chip fixed capacitors (~3.1 pF each) to the touch mode variable capacitor (Fig. 1d). Every switch event leads to a jump in capacitance so that the sensor produces pressure-triggered digital signals.

The key factor to realizing the switch mode function is to create a capacitive cavity without coating it with the material used to seal the cavity in its sacrificial micromachining process (if the cavity surfaces are coated by a sealant film, either dielectric or metal, the function can fail). This requirement is achieved by the micro-Tesla valve-based channels (Fig. 1b). The Tesla valve uses teardrop-shaped loops in a channel to create a unidirectional fluidic flow^35^, which has been commonly used for microfluidic devices^36–38^. We exploit this valve’s one-way function as the first approach to enabling device microfabrication. In particular, on-chip micro-Tesla valves with additional unique channel structures are utilized to effectively release and seal the cavity using a vapor-phase thin-film deposition technique while ensuring that the cavity is free of the sealant film, allowing for ohmic contact between the membrane and individual switch leads. During membrane release, the sacrificial layer is dissolved out of the cavity through the shortest path in the opposite direction of the one-way Tesla valves (Fig. 1b—red). Shortening the path length is crucial for a complete release and preventing residue from becoming trapped within the formed cavity. The created cavity is vacuum sealed by a conformal coating of Parylene C that fully blocks the channels (Fig. 1b—blue). Here, vaporized Parylene molecules travel into the channels in vacuum and pass directly into the multiple Tesla valves and U-turn back into the main path of the channel to oppose the forward molecular flow. This causes Parylene to be trapped by the valves and deposited onto the channel walls to limit the forward flow. Moreover, there are several dead-end paths, referred to in this paper as “sponges”, between those valves; these sponges increase the channel’s inner surface area to ensure that any Parylene that escapes the Tesla valves has a higher probability of adhering to the channel walls before reaching the cavity. Consequently, Parylene C completely blocks the microchannel to achieve vacuum sealing of the sensor cavity.

The predicted performance of the sensor capacitance can be expressed as:

1 $$\begin{array}{l}C_s\left( p \right) = C_{{\mathrm{touch}}}\left( p \right) + C_{{\mathrm{noncontact}}}\left( p \right)\\ \qquad\,\,\,\,\,\,\,\,\,\,\,+\, C_{{\mathrm{switch}}}\left( p \right) + C_{{\mathrm{structural}}}, \end{array}$$

where *C*_*s*_(*p*) is the sensor capacitance as a function of the externally applied pressure, *p*, *C*_touch_(*p*) is the touch mode capacitance from the fixed electrode in contact with the membrane, *C*_noncontact_(*p*) is the rest of the capacitance from the fixed electrode (not in contact with the membrane), *C*_switch_(*p*) is the capacitance from the switch events, and *C*_structural_ is the lumped parasitic capacitance from all the structural parts of the device with the dielectric sandwiched between gold (such as the frame around the membrane). The touch mode capacitance can be calculated by a circular area integral as:

2 $${C_{{\mathrm{touch}}}\left( p \right) = {\int}_0^{\frac{{3\pi }}{2}} {{\int}_0^{r_t\left( p \right)} {\frac{{\varepsilon _d}}{{t_d}}rdrd\theta ,} } }$$

where *r* is the polar radial position on the membrane solved from 0 to *r*_*t*_(*p*), the radial edge of the contact area on the membrane, *θ* is the polar angular position on the membrane solved from 0 to 3*π*/2 radians, that represents 3/4 of the circular membrane (2*π*), *ε*_*d*_ is the dielectric constant of silicon nitride, and *t*_*d*_ is the thickness of silicon nitride. When solved, the touch mode capacitance shows a linear increase in capacitance until the membrane deflection starts to saturate and the capacitance plateaus^1–4^. The switch mode capacitance can be expressed as:

3 $$\begin{array}{*{20}{c}} {C_{{\mathrm{switch}}}\left( p \right) = \mathop {\sum }\limits_0^{n\left( p \right)} \frac{{\varepsilon _dA_{{\mathrm{switch}}}}}{{t_d}}} \end{array},$$

where *A*_switch_ is the area of each of the on-chip fixed capacitors patterned outside of the membrane area and *n*(*p*) is the total number of closed switches in contact with the membrane, which can be calculated with a ceiling function as:

4 $${n\left( p \right) = \Bigg\lceil\frac{{r_t\left( p \right) - x}}{{w_s + s_s}}} \Bigg\rceil,$$

where *x* is the distance from the center of the membrane to the start of the first switch lead, *w*_*s*_ is the switch lead width, and *s*_*s*_ is the space between switch leads. A sum of *w*_*s*_ and *s*_*s*_ represents the switching pitch. The parasitic capacitance can be described as:

5 $$\begin{array}{*{20}{c}} {C_{{\mathrm{structural}}} = \frac{{\varepsilon _dA_{{\mathrm{structural}}}}}{{t_d}}} \end{array}$$

where *A*_structural_ is the total area from the structural parts of the device that cause this invariable capacitance. The capacitance *C*_noncontact_(*p*) is considered negligible compared to *C*_touch_(*p*), as the vacuum gap is significantly larger than the nitride thickness that forms the touch mode capacitance (a more detailed model for the cavity capacitance can be found elsewhere^4^).

**Fig. 2:**
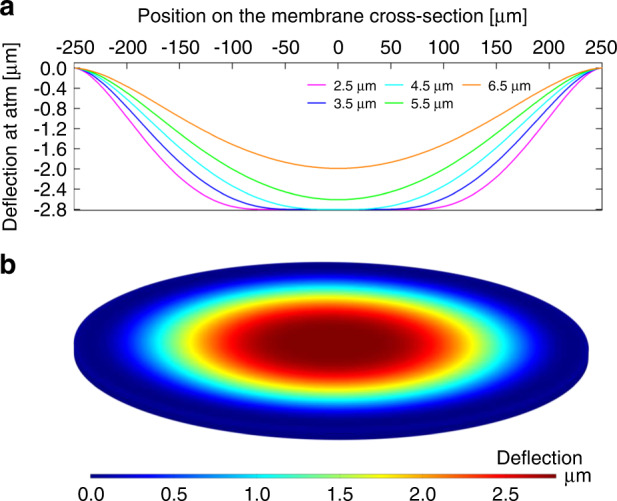
Simulated deflections of the sensor membrane. **a** 2D cross-sections of the membrane with varying gold thicknesses and a fixed 4.6-µm Parylene thickness with a vacuum-sealed cavity at ambient atmospheric pressure. The membrane is constrained using a contact pair set as the bottom of the vacuum cavity within the sensor at 2.8 µm (sacrificial layer thickness) underneath the membrane. **b** A 3D profile simulation of the membrane with 3-µm thick gold at atmospheric pressure.

The membrane mechanics can be analytically modeled to predict its deflection in response to an applied pressure under an assumption of small displacements, as described in the literature^1–4^. The touch mode operation, however, involves relatively large deflections of the membrane, and thus its nonlinear behavior makes finite element methods (FEM) better suited to predict the membrane’s deflection and resultant contact with the substrate. Accordingly, a FEM model was developed using COMSOL Multiphysics^®^ (version 6.0) to simulate the static touch mode conditions. The model assumes that the sensor is symmetric and stictionless with a plain/flat rigid bottom surface for simplicity. Table 1 shows the sensor’s design parameters chosen for this simulation as well as for the prototype fabrication (next section). A bilayer of gold (electroplated, with a thin titanium adhesion film) and Parylene C sealing/packaging film constitutes the membrane, whose thickness is varied by that of the gold layer to adjust the mechanical flexibility of the membrane. The simulated deflection was compared to the preliminary experimental result from a test membrane structure (with a 6-µm gold thickness) measured using white light interferometry (WLI; Profilm 3D, Filmetrics, CA, USA), and the findings suggested that the fabricated membrane had a tensile stress of 26 MPa. This residual stress is applied in all the modeled membrane analyses. Sample simulation results of the membrane deflection with varying gold thicknesses (2.5–6.5 µm) are displayed in Fig. 2. This analysis indicates that the membrane touches down on the bottom at atmospheric pressure when the gold thickness is ≤5.2 µm, which is consequently targeted in the prototyped gold electroplating process. The current device is designed to accommodate a total of 29 switch leads to ensure that they are available for contact, irrespective of the fabricated membrane thickness.

**Table 1:** Design parameters of structural dimensions and related material properties (σ: stress, E: Young’s modulus, ν: Poisson’s ratio)

Parameter	Size [µm]	Relevant parameters
**Sensor Chip**		
Width	870	
Length	3200	
Substrate thickness	500	
**Membrane**		σ 26 MPa
Radius	250	
Electroplated Au thickness	2.5 to 6.5^a^	E 78 GPa, ν 0.44
Ti (adhesion layer) thickness	0.020	E 116 GPa, ν 0.32
Parylene thickness	4.6	E 2.76 GPa, ν 0.40
**Sensor Gap (before Sealing)**	2.8	
**Fixed Au Electrode**		
Si_3_N_4_ thickness	0.275	ε 9.7^b^
Au thickness	0.11	
Substrate SiO_2_ thickness	1.5	
**Au Switch Leads**		
Width	4	
Spacing	4	
Offset from center	4	
**Fixed Switch Capacitors**		C ~3.12 pF/Switch
Length	50	
Width	200	
Si_3_N_4_ thickness	0.275	ε 9.7^b^
**Release/Seal Channels**		4 Channels/Cavity
Overall length	178	
Overall width	420	
Channel entrance width	10	

^a^The fabricated sensor had a 2.85-µm thickness.

^b^The constant was experimentally measured with the capacitance and known area of characterization structures.

## Sensor fabrication

**Fig. 3:**
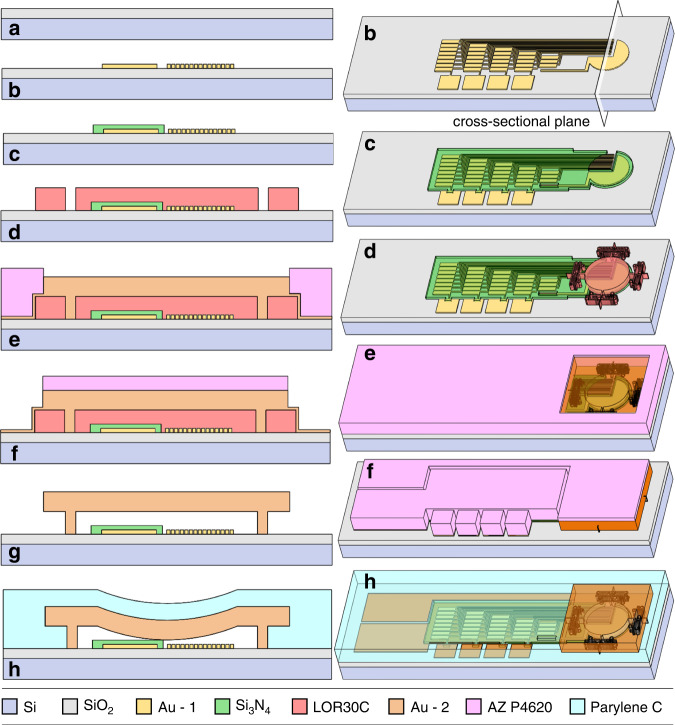
Microfabrication process flow. **a**–**h** Cross-sectional views of the device under processing (left) and the corresponding 3D views (right). The cross-sections are made along the white perpendicular plane shown in the 3D view in **b**. The illustrated dimensions are not to scale. Gold constitutes two separate layers (as shown with different colors) for the evaporated substrate electrode (Au - 1) and the electroplated membrane layer (Au - 2).

The sensor prototype is fabricated through surface micromachining onto a silicon wafer (4 inch, 500-µm thick, *p*-type, <100>, 1–10 ohm·cm) with 1.5-µm wet thermal oxide (Fig. 3a). The process flow uses five patterning steps with ultraviolet (UV) light lithography performed using a maskless aligner (MLA 150, Heidelberg, Germany). First, the fixed electrode layer is deposited using Ti/Au e-beam evaporation (10/100 nm) and patterned at 4-µm resolution with a bilayer lift-off process^39^ using LOR3A (425 nm baked at 200 °C for 5 min) and S1813 (1.6 µm soft baked at 115 °C for 1 min) photoresists (Fig. 3b). Briefly, bilayer lift-off works by undercutting LOR3A as it isotopically dissolves during S1813 development^39^. Next, a 275-nm Si_3_N_4_ film is deposited using plasma-enhanced chemical vapor deposition at 284 °C and patterned using an S1813 photoresist (1.6 µm soft baked at 115 °C for 1 min) and a CF_4_/O_2_ reactive ion etching timed to minimize etching of the thermal oxide underneath (Fig. 3c). The sacrificial layer is then formed by spin coating a bilayer of LOR30C (2.8 µm baked at 170 °C for 5 min) and S1805 (400 nm baked at 115 °C for 1 min) photoresists using a reported process^40^. The bilayer is UV exposed and developed upside down to ensure that the LOR30C undercut has a positive sidewall profile underneath S1805 (which is critical to the conformality of a future sputter step). The sacrificial layer is finalized with an acetone dip for 20 sec that dissolves S1805 without affecting LOR30C (Fig. 3d).

The membrane layer is then formed first by sputtering Ti/Au (20/300 nm) to make a conformal coating that seals all the previous layers and protects the LOR30C from a future photoresist development process. This sputtered film later serves as the seed layer for gold electroplating to complete the membrane. To form an electroplating mold, AZ P4620 photoresist is spin-coated to 10 µm and soft baked at 110 °C for 1 min. The photoresist is then UV patterned but not developed and kept in a UV-protected wafer carrier. Afterward, a dicing saw is used to separate the wafer into individual 13 × 13-mm^2^ chips (each containing multiple sensors and other characterization structures; refer to Fig. 5a, b). The dicing process is conducted under yellow light with the undeveloped photoresist acting as a protective layer, which when developed after dicing, will fully remove any diced particle from the critical areas previously UV exposed. The mold is hard-baked to ensure that it survives the electroplating process by ramping temperatures up and down (steps of 65 °C for 2 min, 110 °C for 1 min, and 120 °C for 1 min). Each chip is then electroplated in a bath of potassium aurocyanide (24 K Pure Gold, Gold Plating, UT, USA) with a current density of 26.9 mA/mm^2^ for ~20 min (well shorter than the ~40 min window where the hard-baked mold starts to deteriorate in the bath) to obtain the targeted membrane thickness (Fig. 3e), followed by stripping the electroplating mold with 1165 Remover (at 60 °C).

Afterward, the formed gold layer is patterned to finalize the membrane structure of the sensor and provide access to the LOR30C sacrificial layer channels. Accordingly, a new 6-µm layer of AZ P4620 is patterned on the single chip with a soft bake at 110 °C for 2 min and a hard bake with an up-and-down ramp (in steps of 75 °C for 1 min and 110 °C for 1 min). The new photoresist is used as protection for a timed 2.5-min etch of Au with potassium iodide, followed by a timed 15-s Ti etch with 5% hydrofluoric acid (Fig. 3f). Individual sensors can be optionally separated into microchips before the subsequent membrane release and sealing (to avoid any potential damage to the released structures) by spin coating AZ P4620 and then dicing (Fig. 5b, c). In this step, microchips are diced around their perimeter with a partial depth (70% of the wafer thickness, to secure all microchips during the process) and then manually separated from the 13 × 13-mm^2^ chip.

**Fig. 4:**
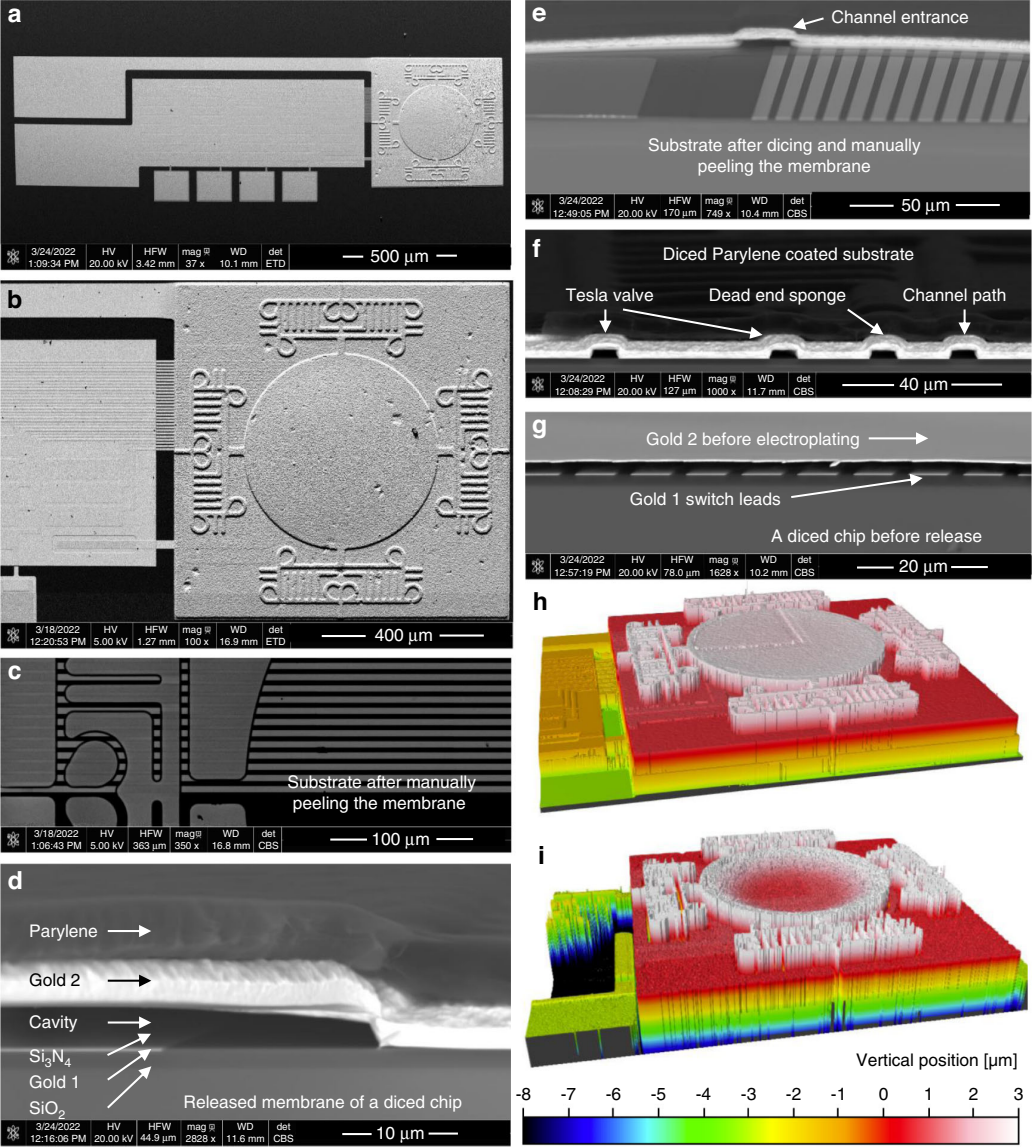
Fabricated device structures imaged by scanning electron microscopy (SEM, a–g) and WLI (h, i). **a** Released membrane before Parylene C sealing showing an entire device. **b** Sensor membrane and micro-Tesla valve channels. **c** Switch leads on the substrate after membrane release (shown by manual peeling of the membrane). **d** A cross-section of the suspended membrane after Parylene sealing. **e** Post-sealing cavity’s internal structures on the substrate (exposed by peeling the membrane) show an array of switch leads and a release channel with no sign of Parylene. **f** A sealed device diced to show cross-sections of a Tesla valve, dead-end sponge, and channel. **g** A diced device before electroplating and release. **h** Measured 3D profile of a released membrane before Parylene sealing showing a flat membrane profile, and **i** after sealing under atmospheric pressure showing a deflected membrane profile.

**Fig. 5:**
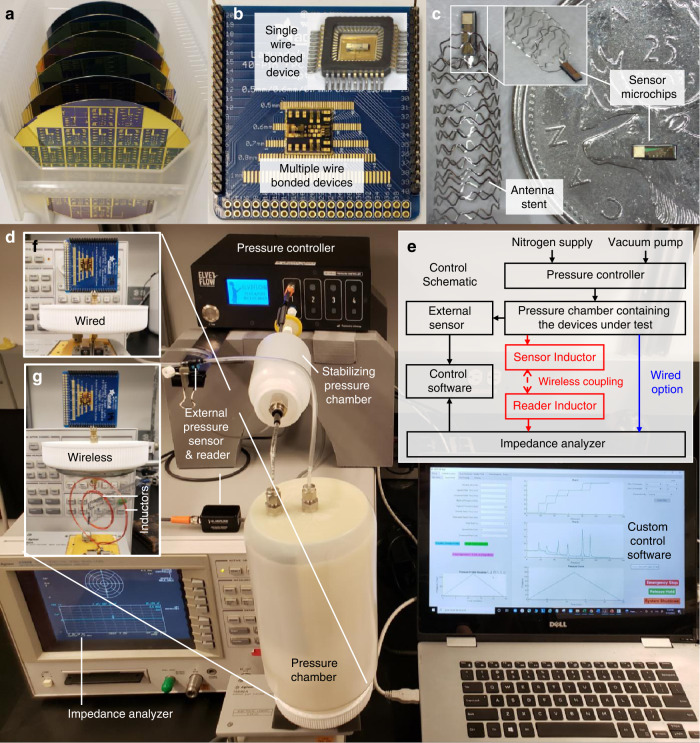
Device samples and experimental setup. **a** Silicon wafers with 400^+^ devices/wafer. **b** Multiple sensor devices on a single chip wire-bonded to a PCB adapter, with an inset showing a diced single device wire-bonded on an integrated-circuit chip carrier. **c** A fabricated microchip of a single sensor on a Canadian quarter coin (for size reference—right) and another sensor microchip on a tab-like platform (~0.6 ×2.0 mm^2^, not visible in the image) of an expanded vascular smart/antenna stent^28,31^ (as a potential application example—left). **d** Pressure testing system with insets showing the control architecture (**e**), as well as the open-chamber configurations used for the wired (**f**) and wireless (**g**) configurations.

Next, a heated (65 °C) mixture of equal parts acetone and 1165 Remover is used to strip the photoresist from the previous steps and to dissolve the LOR30C sacrificial layer through the micro-Tesla valve channels. The addition of acetone, along with the optimized formation of the sacrificial layer, helps dissolve it and mobilize the solution through the release channels. The membrane release process is highly selective and can be run for days at elevated temperatures without causing structural damage. For the current channel design, the chips are left in the solution for 4 days to ensure full membrane release. The chip is then transferred into isopropyl alcohol and dried using a critical point dryer in stasis mode (Fig. 3g), after which the membrane flatness is inspected using WLI (Fig. 4h). The on-chip sensors are then electrically interfaced through wire bonding to gold-plated printed circuit boards (PCBs) or chip carriers (refer to Fig. 5b). Finally, 4.6 µm of Parylene C is conformally coated (PDS 2010, Specialty Coating Systems, IN, USA) in vacuum to package the wired sensors and vacuum seal the cavities by blocking the micro-Tesla valve channels (Fig. 3h). This is checked with WLI at atmospheric pressure by observing the inward deflection of the membranes after transferring the chips out of the coater chamber (Fig. 4i). The fabricated devices are shown in Fig. 4. Destructive inspections indicated that Parylene was absent from the surfaces of the internal cavity and the channels; this was further verified through the ohmic contact events observed during device pressure testing (Results section).

## Wired and wireless experimental protocol

The performance of the fabricated sensors was experimentally assessed in both wired and wireless configurations. The final diced chips wire-bonded to PCBs were tested using a pressure control system with a custom chamber, with which the sensors’ signals were measured in response to elevated gauge pressures (Fig. 5d). The system architecture is illustrated in Fig. 5e. A custom MATLAB^®^ code was used to calibrate and automate the measurement through closed-loop control of the chamber pressure using a commercial pressure controller (OB1-MK3+ with MPS1, ElveFlow, France). The code was also used to collect real-time data from an impedance analyzer (4396B, Agilent Technologies, CA, USA) coupled to the sensor under test through either a wired or wireless interface.

In the wired setting, the PCB was directly connected to the analyzer via a feedthrough connector on the chamber (Fig. 5f). The test frequency (4.5 MHz) was selected to be sufficiently (~30×) lower than the device’s self-resonant frequency to avoid interference in evaluating the impedance and capacitance of the devices. Prior to testing, the membranes were conditioned by cycling gauge pressure (three times) between zero and full-scale pressure (similar to common exercise protocols recommended with commercial pressure sensors for reducing hysteresis^41^). The response measurement of each sensor was repeated three times, each by ramping the gauge pressure up and down at a rate of 10 mmHg/min while recording the capacitance data every 2.5 s (filtered with a 10-Hz bandwidth and 5× averaged).

For wireless testing, the sensor in the chamber was connected to a transmitter coil/antenna (~40-mm diameter, 24 gauge wire, and 930-nH inductance), which was inductively coupled with a reader coil/antenna (~75-mm diameter, 24 gauge wire, and 965-nH inductance) connected to the analyzer for remote tracking of pressure change applied to the sensor (Fig. 5g). The theory of wireless telemetry based on the inductive coupling is well described elsewhere^28–30^. Briefly, the capacitive sensor and the inductive coil together form an inductor-capacitor (LC) tank circuit with the unique resonant frequency, *f*_*s*_, theoretically defined as:

6 $$\begin{array}{*{20}{c}} {f_s(p) = \frac{1}{{2\pi \sqrt {L_sC_s(p)} }},} \end{array}$$

where *L*_*s*_ is the inductance of the transmitter coil^28–30^. The quality factor of the tank circuit, *Q*_*s*_, can be described as:

7 $$\begin{array}{*{20}{c}} {Q_s(p) = \frac{1}{{R_s}}\sqrt {\frac{{L_s}}{{C_s(p)}}} } \end{array}$$

where *R*_*s*_ is the parasitic resistance of the tank circuit^28–30^. The inductive coupling causes a dip in the impedance phase of the reader antenna, Δ*φ*_dip_, whose frequency corresponds to *f*_*s*_ of the tank. An approximate phase dip amount can be expressed as:

8 $$\begin{array}{*{20}{c}} {\Delta \varphi _{{\mathrm{dip}}} \cong \tan ^{ - 1}\left( {\frac{{2\pi f_sM^2}}{{R_sL_r}}} \right) = {{{\mathrm{tan}}}}^{ - 1}\left( {Q_sK^2} \right)} \end{array}$$

where *L*_*r*_ is the inductance of the reader antenna, *M* is the mutual inductance between the tank’s coil and the antenna, and *K* is the coupling coefficient^28–30^. This system enables wireless reading of the tank’s resonant frequency via the phase dip, allowing for tracking of changes in the sensor capacitance induced by varying pressures. The experiments were performed from 0 to 120 mmHg gauge pressure with an 8-mmHg/min ramp rate while reading the phase dip signal (filtered with a 3-kHz bandwidth and 3× averaged) every 2.5 s using the analyzer via the reader antenna powered at 0 dBm.

## Results

**Fig. 6:**
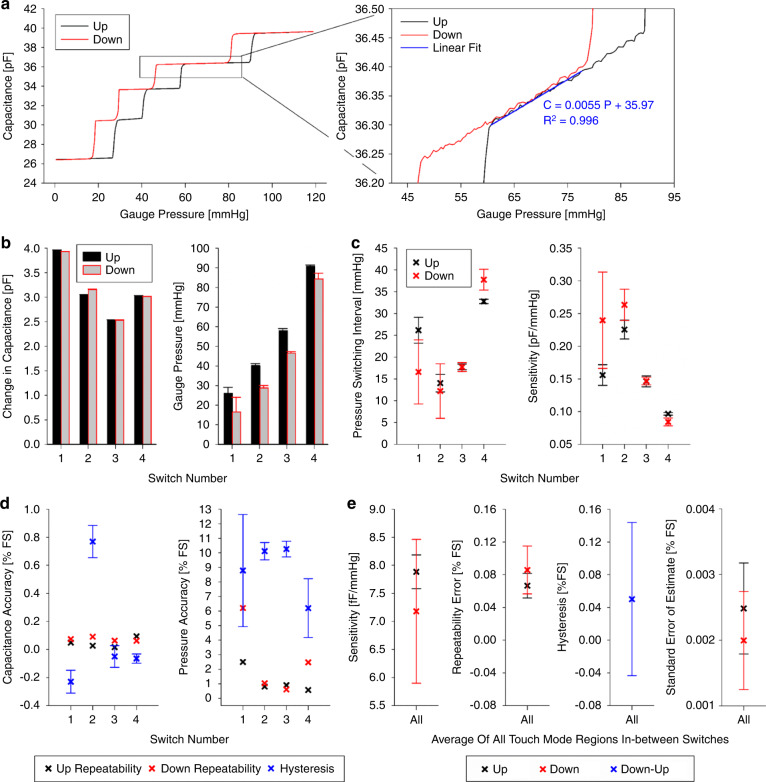
Measurement results from wired tests under varying pressures. **a** Representative plot of the capacitance of the sensor prototype as a function of pressure; the close-up plot shows the regions between switch events with a linear regression fit of the upward data (shown with the associated equation and *R*^2^ value). A summary from three consecutive experiments showing **b** the mean ± the standard deviation (SD) of the switched capacitance and pressure recorded in the up and down trajectories and **c** the switching pressure interval and the sensitivity. **d** The accuracies of the switched capacitance and pressure from three experiments, with the repeatability error as the SD and the hysteresis as the mean ± SD. **e** The touch mode five regions (in-between switch events) characterized from those experiments for the up and down mean ± SD with the sensitivity, repeatability error as the average maximum SD, hysteresis as the average maximum error, and the linearity as the average standard error of estimate for the linear regression in the five regions. The percentages in **d** and **e** are calculated relative to the FS capacitance (13.21 pF) or the FS pressure (120 mmHg).

Through measurements with controlled pressure, the fabricated sensors were demonstrated to provide, as designed, the switch mode function and resultant stepwise capacitive changes, in addition to the touch mode gradual linear increase of the capacitance as a function of applied pressure. This can be seen in Fig. 6a, which displays a representative response of the sensor to applied pressure, showing the two sensing modes while varying pressure up and down.

A summary of the measured switching response is shown in Fig. 6b, c. The baseline capacitance of the fabricated sealed sensors at atmospheric pressure was measured to be 26.40 ± 0.034 pF (SD). The total incremental change had an average of 13.21 pF observed in the sensor’s capacitance for a gauge pressure increase from 0 to 120 mmHg, with 12.57 pF coming from four switch events (2.53–3.96 pF per switch) and the rest of 0.64 pF coming from the touch mode response occurring between the switch events. During the upcycle, those switch events occurred at approximately 26, 40, 58, and 91 mmHg (intervals ranging from 14 to 33 mmHg). During the down cycle, they occurred at approximately 17, 29, 47, and 84 mmHg (intervals ranging from 12 to 38 mmHg). The equivalent capacitive sensitivity of switching, measured as the change in capacitance relative to the switch pressure interval, was calculated as 80–240 fF/mmHg.

The accuracy of the recorded switch events is summarized in Fig. 6d as a function of the measured full-scale (FS) capacitance (13.21 pF) and tested FS pressure (120 mmHg). The repeatability error of the switched capacitance ranged from 0.01 to 0.09%FS, and its hysteresis (deviation between the up and down results) ranged from −0.23 to 0.77%FS. The repeatability error of the switching pressure ranged from 0.6 to 6.2%FS, and its hysteresis ranged from 6.2 to 10.3%FS.

Regarding the performance of the touch mode portion of the sensor, the capacitive response exhibited high linearity (with an average coefficient of determination, *R*^2^, from all five regions between switch events of 0.9933 and 0.9794 for the up and down trajectories, respectively). These regions were quantified for the three repeated consecutive measurements using fitted linear curves (Fig. 6e), from which the sensitivity (the slope of the curve) was found to be 7.53 fF/mmHg on average. The accuracy was quantified as a percentage of the FS capacitance based on the repeatability error that ranged from 0.07 to 0.09%FS, hysteresis that averaged 0.05%FS, and the standard error of the estimate (a measure of the linearity of the in-between regions) that ranged from 0.0020 to 0.0025%FS.

**Fig. 7:**
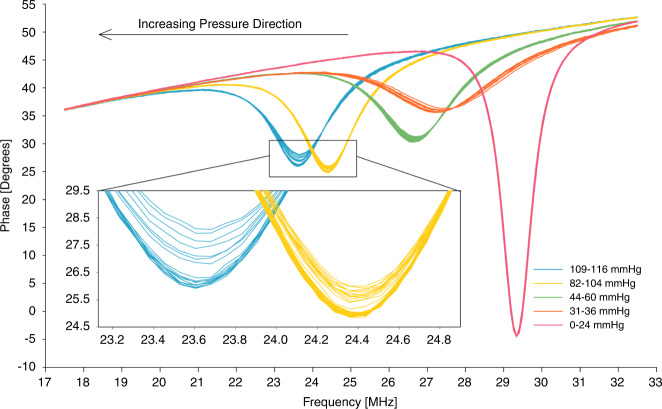
Wireless pressure tracking test. The impedance phase of the reader antenna with a dip caused by the resonance of the sensor’s LC tank was recorded as a function of frequency while varying the applied pressure. The resultant dip frequency shifts between switch events showing switch-induced jumps of the tank’s resonant frequency (five touch mode regions plotted with different colors for the listed pressure ranges). Increasing pressure resulted in lowering the frequency, as theoretically predicted (also shown with an arrow in the plot). The inset displays a close-up of the phase dips in the regions after the third and fourth switch events, each indicating gradual dip frequency shifts with applied pressure.

A sample result of the wireless sensing demonstration is shown in Fig. 7. The baseline frequency of the phase dip caused by the resonance of the sensor’s LC tank at atmospheric pressure was measured to be 29.8 MHz (which matched well with the corresponding frequency of 30.6 MHz measured by direct probing of the same LC tank through a wired interface). As shown, this wirelessly detected dip frequency decreased as the applied pressure rose, owing to an increase in the tank’s capacitance, as theoretically predicted (Eq. 6). Pressurizing to 120 mmHg resulted in the four switch events (demonstrated by showing the five regions surrounding these switch events), as seen in the wired test, with a total frequency shift of 5.76 MHz (0.60–2.19 MHz shift per switch). The equivalent frequency sensitivity of switching (i.e., the change in resonant frequency relative to the switch pressure interval) was calculated as 32.50–101.62 kHz/mmHg. For the touch mode portion of the sensor, the frequency sensitivity in the corresponding regions was measured to be 12.23 kHz/mmHg on average.

## Discussion

The experimental results verified the feasibility of the proposed switch mode capacitive pressure sensor and the effectiveness of its design and microfabrication approach. The sensor demonstrated large abrupt changes in the capacitance (or the resonant frequency in the wireless test) in response to varying ambient pressures, with a significant rise in the capacitive sensitivity. We also showed that the sensor provided the touch mode analog sensing function that was superimposed onto the switch mode function. The addition of the touch mode component enables pressure sensing between switch events.

To assess our sensor’s performance, we compare it to two commercial MEMS capacitive pressure sensors from Murata (SCB10H-B012, Murata Manufacturing, Japan)^5^ and Protron (Absolute Pressure Sensor, Protron Mikrotechnik, Germany)^6^, as well as published touch mode sensors^2,3,16,19,22,25^, all of which are similar in size and pressure range to our sensor. The published sensors had sensitivities ranging from 0.4 to 4.4 fF/mmHg^2,3,16,19,22,25^, while the commercial sensors ranged from 1.3 to 7.3 fF/mmHg with errors less than 0.1%FS^5,6^. Our sensor had two sensitivities stemming from the switch mode and touch mode operation. The switch mode sensitivity ranged from 80 to 240 fF/mmHg (capacitive error less than 0.8%FS, switching pressure repeatability error of 0.6 to 6.2%FS, and hysteresis of 6.2 to 10.3%FS) led by discrete jumps in capacitance with a switching pressure interval of 12–38 mmHg. This indicates that our sensor provided 11–600× greater sensitivity than commercial and previously reported sensors. Furthermore, for pressure sensing between the switch events, the touch mode operation of our sensor provided an average sensitivity of 7.53 fF/mmHg, which is in the upper range of the sensitivities of the abovementioned commercial and published counterparts.

For the wireless demonstration, the phase dip was used to track the resonant frequency (*f*_*s*_) of the sensor’s LC tank. With varying pressure (*p*), the dip changed not only in its frequency due to the change in the sensor capacitance (Eq. 6) with the switch events, but also in the dip amount (Δ*φ*_dip_), which dropped, as seen in Fig. 7. The main source of this outcome is likely that the switch event added a certain contact resistance in series with the switched capacitors. The change in both the sensor’s resistance and capacitance upon switching causes a change in the Q factor, which in turn alters Δ*φ*_dip_ as in Eq. 8 (with a larger impact by the resistance according to Eq. 7). While the first switch may increase the sensor’s resistance as it adds a series resistor (decreasing Q), each consecutive switch would lower the overall resistance as the corresponding resistors are added in parallel (increasing Q). Accordingly, the changes in Δ*φ*_dip_ with increasing pressure in Fig. 7 appear to follow this potential influence of the switch events on the Q factor. Nevertheless, it is clear that each switch led to a significant (MHz-level) shift in *f*_*s*_ owing to the vastly increased sensitivity. This represents a powerful benefit of the proposed sensor for wireless telemetry because it potentially allows one to detect a tiny Δ*p* even with a small Q factor and/or coupling coefficient (*K*) of the system, which makes the reading of Δ*f*_*s*_ difficult when using a typical/touch mode capacitive sensor (similar to the situation of each dip seen in the close-up of Fig. 7).

In switch mode, the prototype was observed to exhibit variations and hysteresis in switching pressures (Fig. 6d). While the exact sources of these behaviors are unclear and will require further systematic analysis, we speculate that the performance was affected by different factors resulting from the complexities in the contact mechanics of the device. In particular, stiction or adhesion forces involved in the membrane–substrate pair with dissimilar materials could have affected the contact/separation dynamics^26,42,43^ and led to those features. The present asymmetric pattern of the bottom electrode and switch leads might have been another source that causes spatial variations in acting forces that could change the overall membrane dynamics. The WLI of the membrane deflection (Fig. 4i), along with the capacitance measurement before and after vacuum sealing, suggested that the sensor likely did not switch in the initial touched region at atmospheric pressure. This observation could be related to two factors. One is the actual topology of the substrate surface having two portions that are not exactly coplanar (switch leads located 165 nm below the silicon nitride surface), potentially requiring more pressure (than atmospheric pressure) for the membrane to touch the leads. The membrane’s back surface that contacts them is titanium (adhesion layer), and its partial oxidation effect could be another factor. The above limitations might be addressed through different paths, including designing switch leads with radial symmetry, introducing additional topography to the bottom surface (e.g., nanopillars^26^) to minimize the surface interactions with the membrane, modifying the process flow to make the gold leads and silicon nitride coplanar, and removing titanium from the membrane contact surface.

Depending on the application, the switch mode capacitive pressure sensors can be optimized and tuned to operate at different pressure ranges, sensitivities, switching thresholds, and overall capacitive performances. The pressure range can be adjusted by designing a membrane structure with different areas and/or thicknesses (by varying the electroplating deposition of gold). The sensitivity can be increased by decreasing the switch lead width and pitch (switching interval), or connecting the switch leads to capacitors that provide greater capacitances outside the membrane (with, e.g., larger electrode areas or thinner/high-k dielectrics if a smaller on-chip footprint is of interest). In certain applications, instead of having a dense array of switch leads, the number of leads and their turn-on pressures can be carefully selected to achieve specific sensing goals. For example, in medical devices, including smart implants, the locations of a few leads under the membrane can be chosen so that the resultant pressure thresholds represent critical conditions of the patient’s diseased organ that should be detected to provide timely needed treatments. Moreover, if only the pressure-driven switch output is needed, the sensor can be designed without the touch mode region (which currently occupies ~75% of the membrane area). For more versatile applications, switches can be leveraged to activate different circuit elements, other than capacitors, in response to sensed pressures.

## Conclusion

The switch mode capacitive pressure sensor has been investigated for the first time. The sensor’s function was demonstrated with proof-of-concept prototypes enabled through novel designs, simulation analysis, surface micromachining techniques, and integration of micro-Tesla valves, showing significant capacitive and frequency responses to pressure input. The sensor provided both a switch mode capacitive signal produced by a pressure-sensitive mechanical switch array embedded in the sensor’s cavity and a touch mode variable capacitance for linear continuous detection of pressure between switching events. Compared to commercial and reported capacitive pressure sensors operating in similar pressure ranges, the developed prototypes exhibited orders-of-magnitude greater sensitivities, 80–240 fF/mmHg, with comparable capacitive accuracy for the tested gauge pressures. Other performance parameters, including response repeatability and hysteresis, were assessed and reported. Furthermore, the wireless demonstration of pressure tracking showed boundless possibilities with this new sensor, exemplified by the MHz-order variation in frequency-based remote pressure monitoring. This new class of sensors can potentially be a platform technology for pressure sensing with high sensitivity, robustness to noise with large signal levels, and simplified signal processing enabled via A/D conversion. The sensor microchip design and packaging can be refined and optimized for specific applications. Switch mode capacitive pressure sensors could open new horizons in a broad range of application fields. Given the abovementioned merits, for example, the sensor shows great promise for smart medical implants and wireless monitoring of in vivo local pressure to alarm patients of critical conditions.
